# Investigation of Helix-Pultruded CFRP Rebar Geometry Variants for Carbon-Reinforced Concrete Structures

**DOI:** 10.3390/polym15153285

**Published:** 2023-08-03

**Authors:** Daniel Wohlfahrt, Hannes Franz Maria Peller, Steffen Müller, Niels Modler, Viktor Mechtcherine

**Affiliations:** 1 Institute of Lightweight Engineering and Polymer Technology, Technische Universität Dresden, Holbeinstraße 3, 01307 Dresden, Germany; daniel.wohlfahrt@tu-dresden.de (D.W.); niels.modler@tu-dresden.de (N.M.); 2 Institute of Construction Materials, Technische Universität Dresden, Georg-Schumann-Straße 7, 01187 Dresden, Germany; steffen.mueller@tu-dresden.de (S.M.); mechtcherine@tu-dresden.de (V.M.)

**Keywords:** carbon–concrete composites, pultrusion, helix rebar, tensile test, pull-out test

## Abstract

Carbon concrete is a new, promising class of materials in the construction industry. This corrosion-resistant reinforcement material leads to a reduction in the concrete cover required for medial shielding. This enables lean construction and the conservation of concrete and energy-intensive cement manufacturing. Bar-type reinforcement is essential for heavily loaded structures. The newly developed helix pultrusion is the first process capable of producing carbon fiber-reinforced polymer (CFRP) reinforcement bars with a topological surface in a single pultrusion process step, with fiber orientation tailored to the specific loads. The manufacturing feasibility and load-bearing capacity were thoroughly tested and compared with other design and process variants. Approaches to increase stiffness and strength while maintaining good concrete anchorage have been presented and fabricated. Tensile testing of the helical rebar variants with a 7.2 mm lead-bearing cross-section was conducted using adapted wedge grips with a 300 mm restraint length. The new helix geometry variants achieved, on average, 40% higher strengths and almost reached the values of the base material. Concrete pull-out tests were carried out to evaluate the bond properties. The helix contour design caused the bar to twist out of the concrete test specimen. Utilizing the Rilem beam test setup, the helical contour bars could also be tested. Compared with the original helix variant, the pull-out forces could be increased from 8.5 kN to up to 22.4 kN, i.e., by a factor of 2.5. It was thus possible to derive a preferred solution that is optimally suited for use in carbon concrete.

## 1. Introduction

The increasing significance of sustainable materials and technologies in construction, driven by climate change concerns, is evident in the European Union’s goal to achieve a climate-neutral and sustainable European Economic Area by 2050. The target is to reduce CO_2_ emissions by at least 55% compared to 1990 levels by 2030 [1]. Concrete production contributes substantial CO_2_ emissions due to the burning of limestone and the chemical conversion process, resulting in approximately 2.7 billion tons of CO_2_ emissions annually, equivalent to roughly 7% of global anthropogenic CO_2_ emissions [2,3,4,5,6,7,8,9]. Furthermore, this exacerbates the environmental impact through the extraction of sand from riverbeds or the seafloor, leading to coastal erosion and harm to ocean fauna [10,11,12,13]. Despite concrete’s brittleness and low tensile strength, its flexible application possibilities and high compressive strength do make it a popular building material. To compensate for those disadvantages, concrete structures are reinforced with so-called rebar structures [14]. Conventional rebar structures are made of steel and require a protective environment to suppress corrosion processes. In this context, the use of carbon concrete and carbon fiber-reinforced polymer (CFRP) reinforcement structures is becoming increasingly important, as this technology has the potential to conserve resources and significantly reduce CO_2_ emissions. Its excellent mechanical and physical behavior such as high tensile strength, stiffness, and chemical resistance, make it a good alternative as a reinforcing material for concrete structures [15]. Compared to conventional reinforced concrete, carbon concrete has the advantage that its reinforcement structures are chemically inert and therefore not corrosive. This leads to the fact that carbon–concrete structures do not require a high concrete covering to protect the reinforcement structures from corrosion [3,16,17,18]. Depending on the shape structure, roughly up to 40% of concrete can be saved by using CFRP concrete reinforcement [19]. Furthermore, carbon concrete has a longer service life compared to conventional reinforced concrete. The combination of material savings and increased service life can significantly reduce the ecological footprint when using carbon concrete [20,21,22].

**Figure 1:**
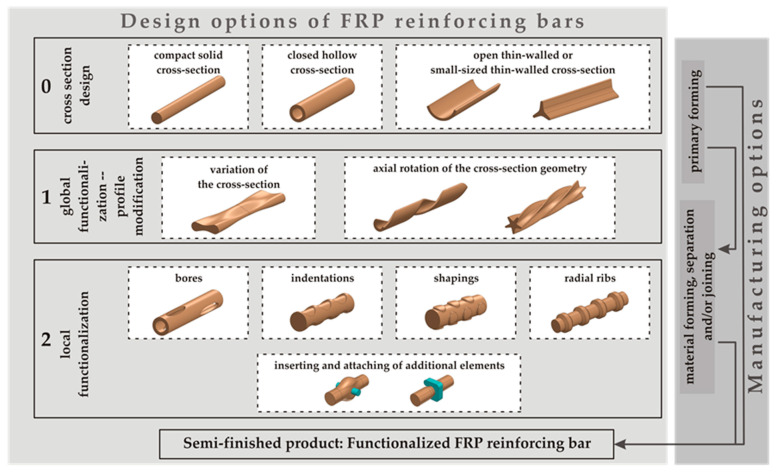
Design and manufacturing options for CFRP rebars with different geometry profiles [19].

In order to support concrete structures under tensile stress, the installed reinforcement structures must have undercuts in the axial direction. This functionalization helps to transfer the tensile stresses acting on the concrete structures to the inner cross-sectional areas of the reinforcement structures [14]. In previous work, design principles for fiber-reinforced polymer reinforcement bars resulting from mechanical stresses were derived based on defined design guidelines. For this purpose, implementation options were systematically developed at various levels. These include not only design variations of the profile cross-section of different cross-sectional designs (Level 0) but also approaches for the global functionalization of bars through axially progressive profile modifications at a global level (Level 1) and local functionalization (Level 2) at the smallest element level. The various design approaches can be seen in Figure 1 [19,23].

Selected functionalization variants were exemplarily implemented in production for a large-scale use of novel carbon reinforcement bars. These were subsequently validated experimentally, further developed geometrically, and the technological and economic implementation was investigated [19,23]. The focus was particularly on achieving necessary characteristic values in construction. An overview of the developed functionalized reinforcement bar variants can be found in Table 1.

**Figure 2:**
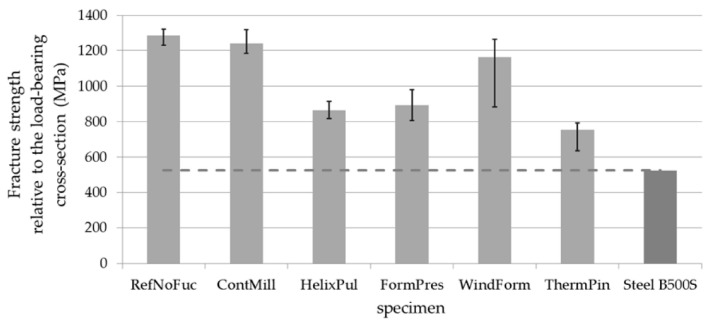
Comparison of the load-bearing capacity of the functionalized CFRP rods compared to the non-functionalized reference rod and conventional steel rebars [19].

The analysis of prototypical functionalized rod profiles (Figure 2), in combination with the evaluation of damage patterns resulting from concrete extraction tests, demonstrated a positive influence of undamaged axially aligned fibers in the load-bearing cross-section. The optimal reinforcement behavior of the reinforcement bars for high reinforcement effectiveness in concrete requires optimal force transmission from the surface area into the entire cross-section of the bar. Shear stresses that occur have components in both axial and radial directions. Ideally, the stiffer and stronger reinforcement fibers, compared to the plastic matrix material, should form this load path. Local fiber reinforcements strengthen the plastic matrix and increase its resistance to the shear failure of the functionalizations.

In terms of these properties, the variants FormPres, ContMill, and HelixPul showed particular advantages among the investigated options. The ThermPin variant allows direct force introduction into the cross-section of the bar through additional elements as a special case. However, these elements significantly reduce the load-bearing cross-section and thus the load capacity. The connection in the WindForm variant is unfavorable for optimal bonding effectiveness due to the short rib width and a fiber orientation almost perpendicular to the loading direction in the functionalization. In comparison, the ContMill variant with sufficient rib width and axial fiber orientation exhibited good load-bearing behavior even in combination with concrete. 

The evaluation of tensile tests, damage patterns, and potential economic feasibility of each production technology formed the basis for selecting the optimization variant for load-appropriate rod functionalizations with a thermoplastic matrix for rod-reinforcement structures. The unique design possibility of the helix contour (HelixPul), as the first CFRP rebar design to be based on an economic single-step pultrusion process, showed high potential for further development and effective use in construction and was thus selected as the preferred option for further investigation [19,23,24].

The application of pultruded fiber-reinforced polymers (FRPs) in the construction sector has a well-established history. Vedernikov et al. [25] reviewed present areas where elements manufactured through pultrusion processes are utilized, with an emphasis on identifying potential avenues for future research.

Zhu et al. [26] experimentally investigated the mechanical performance of glass fiber-reinforced polymer (GFRP) and CFRP rebars by performing uniaxial tensile tests and using a non-contact three-dimensional digital image correlation (3D-DIC) technique for strain measurements, considering the spiral curved surface of the rebars. They found a linear relationship between stress and strain without a yielding stage during the tensile process for both GFRP and CFRP specimens. Additionally, they described the advantages of using 3D-DIC over a clip-on extensometer.

Yun et al. [15] characterized sand-coated CFRP rebars in terms of tensile strength, compressive strength, shear strength, and Young’s modulus. The sand coating is applied to enhance the adhesion between the CFRP rebars and the concrete matrix. It was reported that the tensile strength of the rebars is dependent on their diameter due to the shear lag effect.

Benmokrane et al. [27], inter alia, examined the bond strength between FRP rods and cement grout, as well as their pull-out behavior. It was discovered that, in addition to the properties of the filling grout and the stiffness of the host medium, the surface geometry of the rebars also influences their pull-out behavior.

To further investigate the influence of the rebars’ surface geometry on their mechanical properties and the bond strength between the rebars and the concrete matrix, selected parameters of helix-pultruded carbon-reinforced rebars, such as the pitch of the helix contour and their number, have been varied. The structures were then investigated by performing tensile testing and extraction tests.

## 2. Materials and Methods

### 2.1. Helix-Pultruded Rebars

#### 2.1.1. Carbon Tape Workpiece

Tape-shaped workpieces (SGL-CF-PA6-Tape) from the manufacturer SGL TECHNOLOGIES GmbH, Meitingen, Germany were used for the production of the helically pultruded CFRP-reinforced rebars. These consist of a thermoplastic polyamide 6 matrix, reinforced with SIGRAFIL R C T50-4.0/240-T140 carbon fibers from the same manufacturer, and have a fiber volume content of 45%. They were characterized by tensile tests, and the determined values were compared with the partially available characteristic values from the manufacturer’s datasheets (see Table 2). It was found that the tensile modulus is at a similar level, but the measured tensile strength appears to be lower than the theoretical values in the datasheet. 

#### 2.1.2. Helix-Pultruded Rebar Geometry Variants

In order to further develop functionalized HelixPul rod structures, various geometric parameters were defined, which were analyzed and evaluated in terms of production and experimental considerations through parameter investigations. The following parameters were varied for the investigations:

- Number of helix ribs *n*;
- Pitch *P*;
- Circumferential diameter *D_p_*;
- Equivalent diameter *D_e_*;
- Projected flank area *A_f_*;
- *A_f_/A_i_*.

The area *A_i_* corresponds to the area of the inner circle with diameter *D_i_,* and the projected flank area *A_f_* is obtained from:

(1) $$A_{f}=\frac{\pi }{4}(D_{p}^{2}-D_{i}^{2})$$

**Figure 3:**
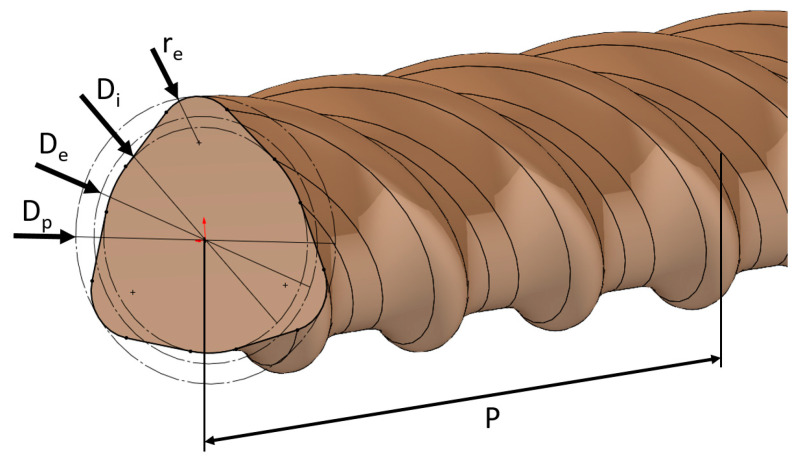
Graphical illustration of the diameters *D_i_*, *D_e_*, and *D_p_* and pitch *P* on the helix-pultruded rebar variant H81.

The corresponding diameters *D_i_*, *D_e_*, *D_P,_* and the pitch *P* are graphically illustrated for the HelixPul variant H81 in Figure 3.

**Figure 4:**
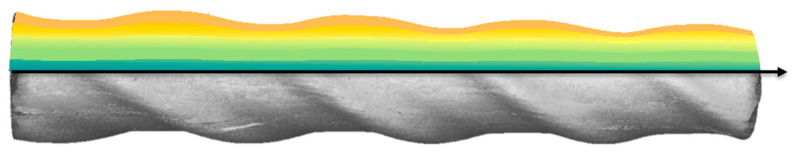
Schematic illustration of the derivation of the local undulation. Whereas red represents strong undulations and green represents weak undulations.

The necessary geometry of the rod functionalization for the composite effect between the reinforcing structure and the concrete matrix requires a strong undulation, especially of the outer fiber layers, which extends into the center of the cross-section of the reinforcing bars and decreases in intensity from the outside to the inside. This relationship is schematically illustrated in Figure 4. These undulations in the load-bearing cross-section reduce the load-bearing capacity and stiffness in the ideal axial direction accordingly. It had to be taken into account that a significant profiling of the reinforcing bars is necessary for the composite effect with the concrete matrix for force transmission. To ensure the good tensile properties of the bars, a nearly undulation-free straight fiber layer is necessary.

The focus of the further development of the reinforcement bars was therefore on creating a balanced ratio between the outer bar profiling and inner straight layers, in order to achieve a compromise between good bond behavior with the concrete matrix and high stiffness. Carbon reinforcement bars were therefore investigated and further developed not only with respect to their tensile properties but also their bond behavior. Due to manufacturing constraints, the realization of the geometry parameters can only be indirectly achieved through external shaping. Therefore, the control of the inner fiber layers is only possible indirectly through the outer contour design and the pultrusion process.

In order to ensure good comparability in terms of assessing the load-bearing capacity of the reinforcement structures, the supporting continuous and undisturbed cross-section of the design variant H81 was adopted as a reference value for all other rebar variants, and an inner diameter *D_i_* of 7.3 mm was defined. Due to the rotational manufacturing technology of the helix-pultrusion process, a circular basic structure of the reinforcement bars is given, around which the functionalization winds spirally in the form of rib-like attachments. The variations of the geometry parameters are summarized in Table 3 along with a graphical representation of the resulting structures.

The variation in the number of ribs over a defined length was influenced by the pitch of the helix, which also affects the orientation of the ribs. The smaller the pitch, the larger the angle of inclination of the ribs. However, since increasing the number of ribs over a defined length reduces the load-bearing potential of the individual ribs in the composite, narrowing the ribs does not necessarily represent an improvement. Therefore, for the design variant H82, the pitch *P* was increased. Insights from preliminary tests showed that the rib height could be lower for a lower fiber undulation and that the undercuts were still sufficiently formed for force transmission in the carbon–concrete composite. In variant H83, the rib height was therefore halved compared to variant H81. This change simultaneously affects the circumferential diameter *D_p_*, the projected flank area *A_f_*, and the ratio of *A_f_/A_i_*, thus affecting three parameters at the same time. The variation in the number of ribs also leads to a change in rib height, as the ribs must be geometrically narrower. If the rib flank angle is kept constant, a lower rib height automatically results. Therefore, in variant H84, the number of ribs was increased to six, resulting in the most delicate rib structure of all variants. Conversely, when reducing the number of ribs from three to two, as in variant H85, there is also an adjustment possibility with respect to rib width. Compared to a narrow rib, a wider rib can significantly better support itself under load. Due to the greatest potential for improvement and influence, variants H83, H84, and H85 were selected and implemented for manufacturing, material, and composite investigations in addition to variant H81, to test the bar geometries of helix-pultruded carbon reinforcement bars.

### 2.2. Tensile Testing of Helix-Pultruded Rebar Elements

The material characterization using tensile testing was performed on the developed and manufactured HelixPul variants H81, H83, H84, and H85. The test was carried out according to the standards for conventional reinforcement systems: EN ISO 15630-1, DIN EN ISO 7500-1, and DIN EN ISO 6892-1 [30,31,32]. Due to the functionalization and the resulting non-uniform surface geometries of the reinforcement bars, the challenge mainly consisted of developing a clamping mechanism that enables the fast and efficient clamping of bar specimens with geometrically free-shaped surfaces without having to make separate preparations for each bar geometry. However, the aforementioned standards only generally describe that the clamping devices must be suitable for testing, and, except for the proposal of different clamping methods, no specification is provided.

**Figure 5:**
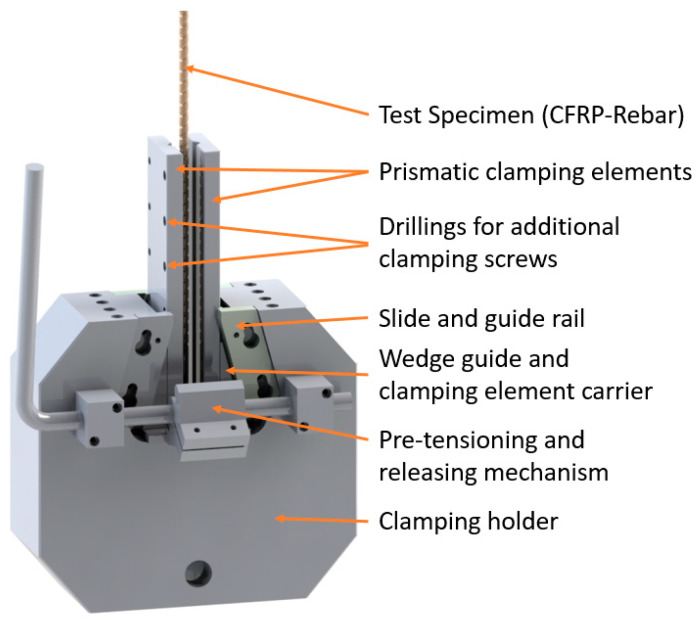
Clamping mechanism for efficient testing of CFRP rebars.

After conceptualizing and evaluating various reinforcement-bar clamping devices, a clamping mechanism based on a wedge clamping system was developed (see Figure 5). The clamping forces can be increased as needed by screw clamping elements. For optimal load introduction, the largest possible clamping range was realized, characterized by a high overlap angle between clamping and cylindrical test specimens. At the same time, the largest possible clamping diameter range was implemented.

The larger the prism opening angle, the larger the clamping diameter of the bar. According to DIN 2214 for the execution of test prisms, cutting angles of 90° and 108° are suggested. A cutting angle of 108° forms a good compromise for clamping the reference bars and covering different bar diameters and was therefore chosen for the construction of the clamping device.

For optimal clamping and load introduction, as well as for the protection of the test specimen from excessively high local clamping forces, prismatic clamping elements are equipped with a compensating layer made of elastomer for testing. The elastomer used is a nitrile rubber from the manufacturer Misumi, designated NB270n-10-300-300. The intermediate layer strips used had the dimensions of 2 mm × 12 mm × 300 mm. Due to the high stress on the compensating material, it cannot be reused multiple times.

All tests were carried out at a standard test climate of 23 °C ambient temperature and 50% relative humidity. The testing machine used was a Zwick Z250 with a testing speed of 2 mm/min. Furthermore, the tensile forces occurring during the test were measured and recorded using a 250 kN force transducer, and the resulting deformations were measured using a contact macro extensometer. 

The specimens used in the study had an overall length of 80 cm, consisting of a free length of 20 cm and an anchoring length of two times 30 cm. The anchored length of the specimens was further secured using screws to fasten the prismatic clamping elements. The screws were tightened progressively from the outside to the inside with stepped torque, beginning at 60 Nm and decreasing by 5 Nm for each subsequent screw to minimize stiffness fluctuations. Afterwards, the rebars were subjected to a load of 20 kN, and subsequently, the screws were retightened. The external screws were tightened up to 80 Nm, while the internal ones were tightened with 2 Nm less each. In case the specimens slipped at higher loads, the test was paused, and the screws were retightened following the specified scheme.

**Figure 6:**
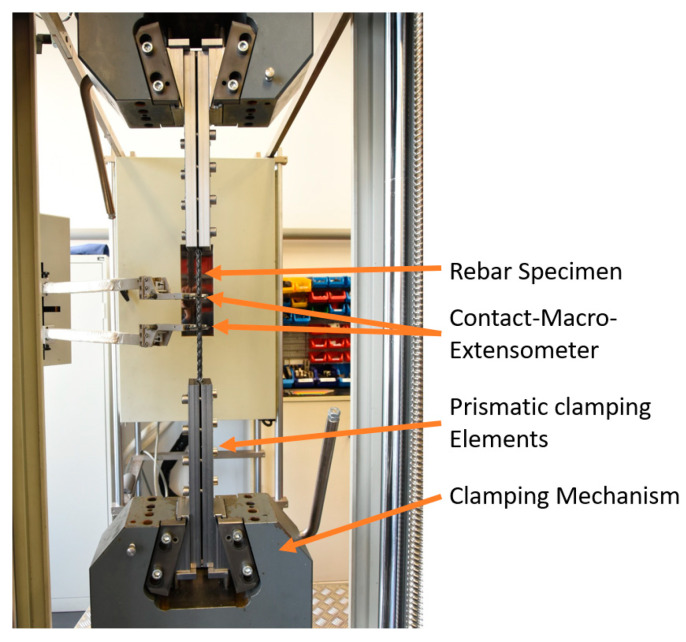
Tensile test setup.

The testing setup used can be seen in Figure 6. The determined characteristic values are the fracture force *F_max_*, fracture strain *ε_max_*, and fracture stress *σ_max_*, which are obtained from:

(2) $$\sigma_{max}=\frac{F_{Bruch}}{S_{0}}$$

where *S_0_* is the cross-sectional area of the bar in the direction of tension. Six specimens were tested for each bar configuration to obtain valid results.

### 2.3. Pull-Out Testing of Helix-Pultruded Rebar Elements

**Figure 7:**
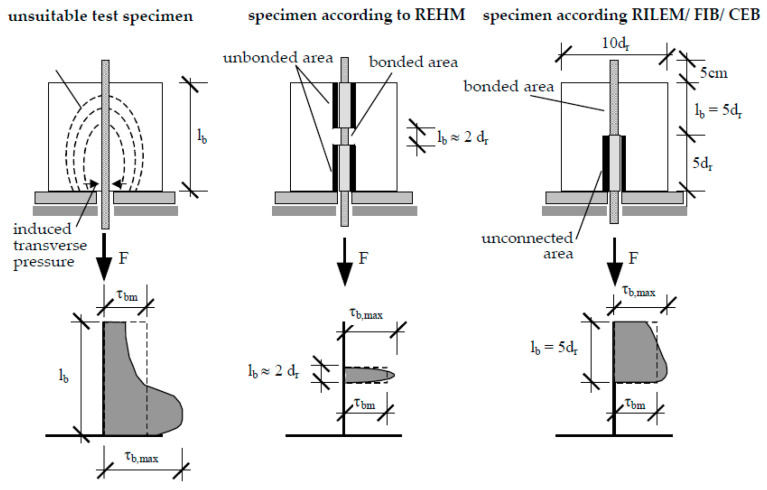
Influence of bond length on the distribution of local bond stresses [33].

In addition to the evaluation of the pure strength and durability parameters, the reinforcement elements should also exhibit sufficient bond strength to the surrounding clay matrix. This is determined in pull-out tests. A specimen shape was selected that allows measurement results in the direct test procedure. Based on the experience according to [33], it is based on the recommendations of RILEM/ FIB/ CEB [34] and the specimen shape according to REHM [35]. Both of these specimens allow measurements in an undisturbed area of the specimen, unaffected by the support. Furthermore, the bond length was reduced to ensure a constant stress distribution over the bar length to be tested (see also Figure 7).

The selected test specimen allows the adaptation of different bond lengths. In order to map a representative range of composite properties, the bond length had to capture the bar geometry peculiarities (e.g., a rotation of the helix in the bar) at least one time. The HelixPul test specimens investigated have the same pitch and thus the same bond length. Testing of the bond properties was carried out on a self-compacting concrete with 5 mm crushed granite grit as the largest aggregate. Accompanying compressive strength and flexural tensile tests showed a compressive strength of approx. 100 MPa and a flexural tensile strength of approx. 5 MPa for this formulation. 

**Figure 8:**
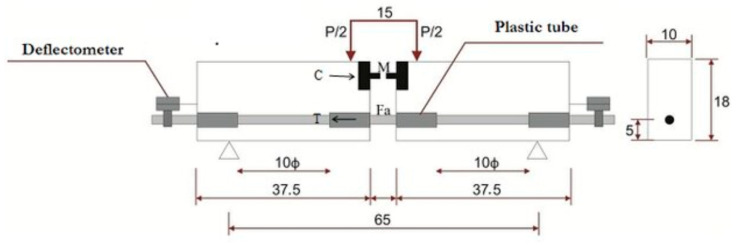
Beam test (test setup according to RILEM) [36].

The present HelixPul bar geometry provides a significant input of torsional energy into the concrete body. Due to this high torsional force input, the RILEM RC5 test was applied as an established test method with more massive component measurements. The test is basically a four-point bending test with a fixed pivot point. The latter absorbs the resulting compressive forces, whereas the embedded bar has to absorb the tensile components. In this case, an equal tensile force is generated on both sides of the bar and a pull-out will occur on the side of the weaker composite. The basic setup of the test is shown in Figure 8.

**Figure 9:**
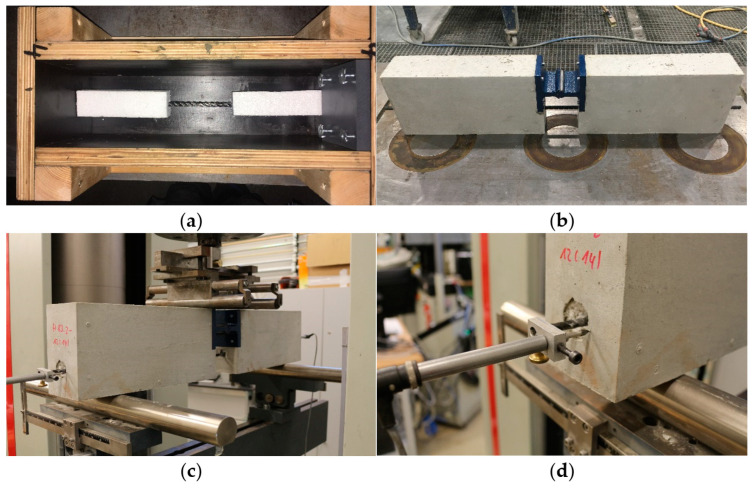
Details of the RILEM-RC5 beam test: (**a**) Prepared formwork with polystyrene decoupling elements; (**b**) Specimen out of the formwork; (**c**) Specimen in the testing machine; (**d**) Detail of pull-out receptor.

Since the carbon rods have a radially lower stiffness than comparable steel rods, a larger-volume Styrofoam insert replaced the recommended plastic sheathing to allow greater bending freedom. Figure 9 shows the production, specimen, and test details of the composite specimens.

For specimen fabrication, the rebar elements were inserted into the Styrofoam elements and positioned in the formwork. In addition, the auxiliary joint was also positioned in the formwork at the intended location to realize a defined pivot point. The formwork was filled with concrete, and the finished specimen was demolded after concrete consolidation. The rebar ends overhanging from the concrete were fitted with an inductive displacement transducer, and the specimens were positioned as a unit in the test fixture.

## 3. Results

### 3.1. Tensile Tests

The results of the tensile tests conducted on the tested HelixPul geometry variants H81, H83, H84, and H85 are summarized in Table 4. In addition to the mean value $\overset{-}{x}$ of the results, the standard deviation *s,* and the relative standard deviation *ν* are also given.

After testing, the characterized reinforcing bars exhibited a valid failure pattern in the area of the free length. This rules out the possibility that clamping effects, such as transverse forces from clamping, were the cause of damage and thus had a reducing effect on the mechanical properties.

**Figure 10:**
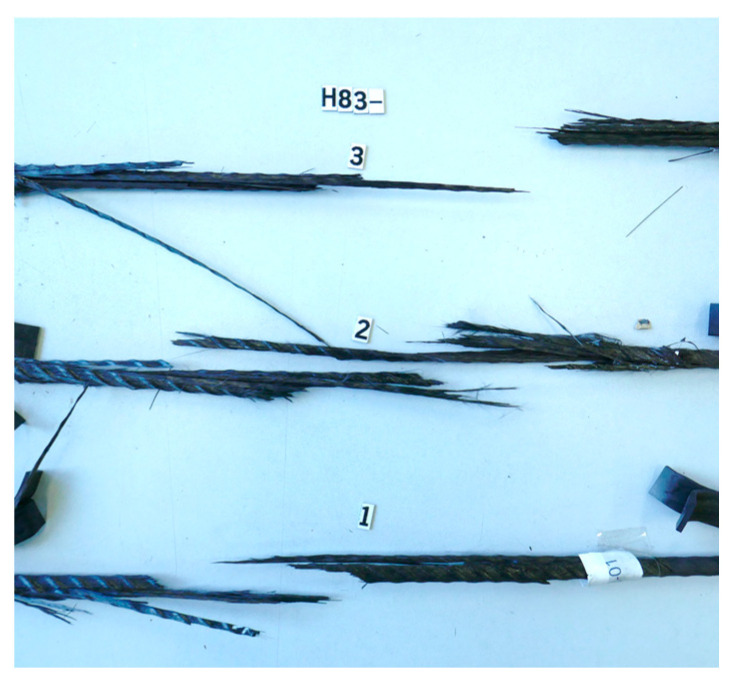
Images of failure using H83 as an example.

All experiments also showed a failure behavior typical for axially reinforced plastics. This is characterized by axial splintering or delamination in combination with uneven failure in the direction of tension. In the manufacturing process, the fibers are pressed into shape slightly under tension, as the matrix material solidifies quickly. This freezes residual stresses, which become visible in the failure shown in Figure 10.

**Figure 11:**
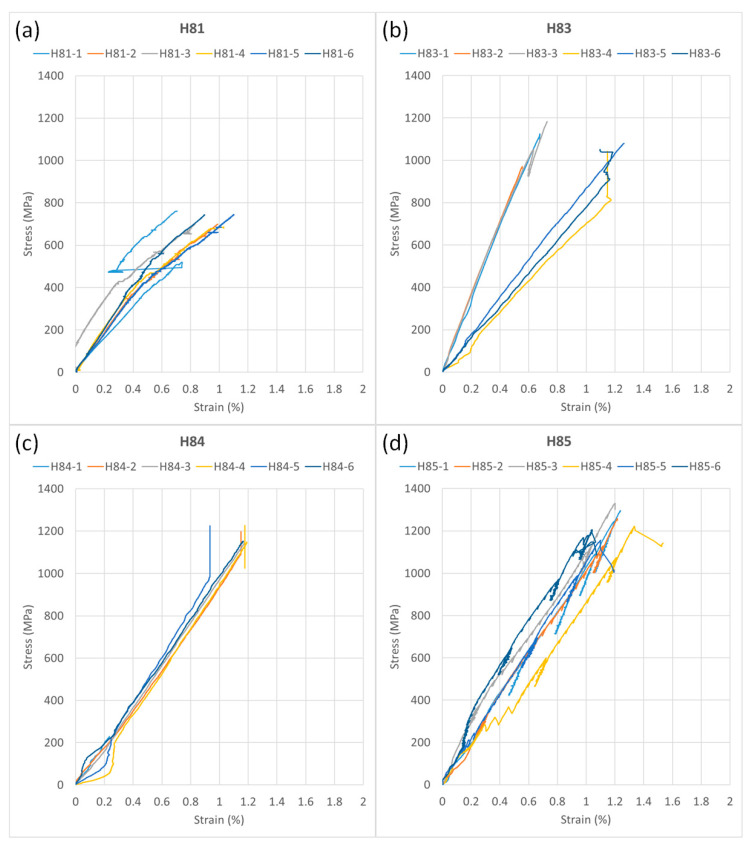
Strain–stress curve of CFRP tensile tests of the HelixPul rebar variants H81 (**a**), H83 (**b**), H84 (**c**) and H85 (**d**).

Due to the different jacket geometries of the reinforcing bars, the contact macro extensometer occasionally slipped during testing, leading to distortions of the strain measurements, which are evident as jumps in the measurement curves (Figure 11).

### 3.2. Pull-Out Tests

In the pull-out test, the bar variants of HelixPul H81, H83 and H84 were tested. The measured maximum pull-out force *F_max_* is related to the respective applied bond length, and thus the characteristic value bond flux *k_max_* (N/mm) is generated. The results of the pull-out test are shown in Table 5.

As already described in Section 2.3, the tests showed that the HelixPul bar geometry leads to a considerable input of torsional energy into the concrete body. Thus, the pull-out test of HelixPul H85 was deliberately omitted, and alternative test options were selected.

**Figure 12:**
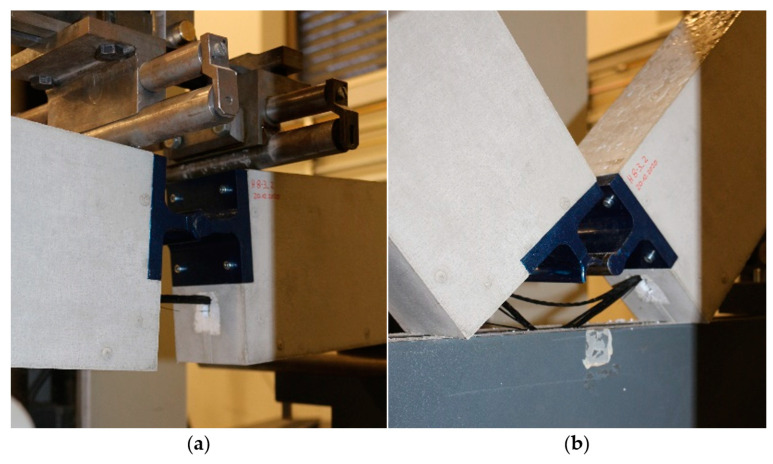
Test details of the RILEM-RC5 beam test: (**a**) Test and significant deflection; (**b**) Failed specimen.

The RILEM test method described in Section 2.3 was used as the RC5 beam test variant seemed suitable for this purpose. Details of the test procedure are shown in Figure 12.

The individual results with the maximum force *F_max_*, deformation *∆l_max_* and deflection *fmax* from the beam tests are summarized in Table 6. During all tests, the force builds up uniformly until the bars are completely detached from the concrete, and the bars begin to move into the middle area of the specimens. The influence of the different Helix variants can be clearly seen. For HelixPul H81, for example, low bond forces are built up. The HelixPul H83 and H85 variants in particular generate significantly higher bond forces. The test setup also confirmed a significantly lower torsional tendency of the reinforcing bars due to the helix contour compared to the single-sided pull-out specimens. However, the bending test specimens require a significantly higher handling effort and material input compared to the pull-out test specimens.

## 4. Discussion

The test results obtained in Section 3.1 are based on the clamping device developed and presented in Section 2.2. This device is intended to facilitate direct tensile testing without the need for the special preparation of the test specimens or the fixture. Additionally, it enables the loading of the bars to tensile failure in the free test length without initiating failure through the clamping area. This achievement was implemented through the design measures described in Section 2.2. Furthermore, tests were conducted to examine the compression-shear behavior of selected elastic clamping materials, including nitrile rubber (NBR), fluororubber (FPM), natural rubber (NR), chloroprene rubber (CR), polyurethane rubber (PUR), plywood, and leather. Among these materials, NBR showed the most promising results. Unfortunately, none of the materials exhibited sufficiently high compression-shear resistance to be suitable for more than one test application.

The tensile tests conducted to characterize the manufactured tension rod variants H81, H83, H84, and H85 from HelixPul production confirmed the suitability of the restraint principle for profiled rod geometries. The test specimens could be clamped sufficiently firmly and without damage. However, settlement effects of the NBR material occurred in some cases, which could be compensated for by manual re-clamping. For efficient and partially automated testing, it is recommended to use a controllable clamping device, such as one that can be realized through hydraulic force application.

**Figure 13:**
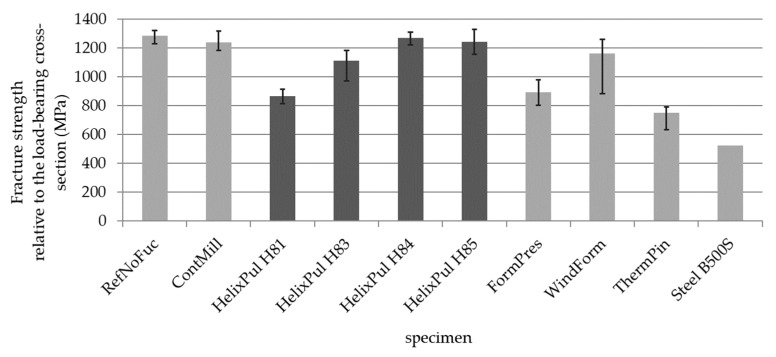
Supplemented tensile fracture stresses of differently functionalized bar test specimens.

Figure 13 depicts the tensile strengths determined, alongside the previous test results. The initial series of tests represent the strengths of unfunctionalized bars, indicating the strength of the base material. Notably, all bars exhibit higher strength, meeting the requirements specified by the DIN 488 or EN 10080 standards for steel reinforcements. As a result, they confirm their suitability as an alternative reinforcement material. During the first series of tests, it became evident that decreasing strength correlates with increasing fiber undulation in the reinforcing bar. This correlation was also observed in the new series of tests on helix-pultruded rebars (HelixPul). Table 7 presents the individual strengths in relation to the base material, as measured using the reference bar. It is evident that halving the rib height (as seen in the comparison of HelixPul H81 with HelixPul H83) almost halves the dropped strength. The variant HelixPul H84, featuring a very low rib height due to the presence of a large number of ribs, achieves nearly the basic strength. Additionally, the HelixPul H85 variant achieves only a slightly lower strength than the HelixPul H84 variant and exhibits a similar strength to the ContMill variant. These findings demonstrate that load-bearing reinforcing bars can be effectively produced using helix pultrusion.

To assess the composite tests, morphological analyses were also carried out to examine the failure mechanisms during the pull-out test and the microstructure of the interphase. These optical observations, in addition to the mechanical parameters, offer insights into the prevailing damage mechanisms and potential improvement options. The observation levels can be adjusted as required, ranging from simple visual inspection with the eye/loupe and extending to more advanced techniques such as light microscopy or scanning electron microscopy. During the pull-out tests, the optical findings revealed minimal adhesion of the concrete matrix to the rebar, and no detectable carbon fiber residues were observed in the pull-out channel. Consequently, the investigations focused on utilizing high-resolution photographs and light microscopic images. 

**Figure 14:**
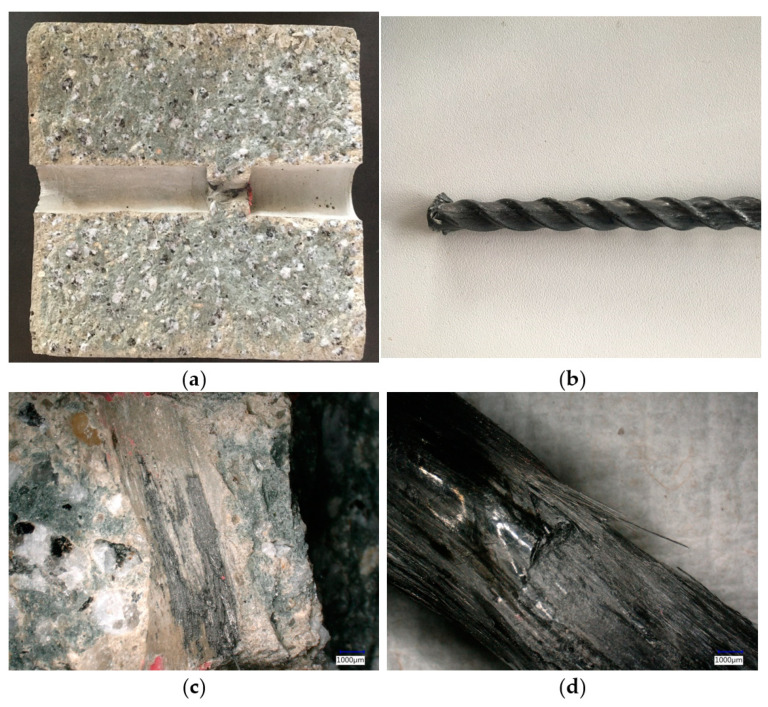
Details of pull-out test specimens: (**a**) HelixPul H81—split concrete part of the pull-out test specimen after the pull-out test; (**b**) HelixPul H81—pulled-out probationary bar without visible change of the surface; (**c**) HelixPul H83—adhesions from CFRP of the bar to concrete matrix; (**d**) HelixPul H83—damages on the pulled-out bar.

The pull-out test specimens of HelixPul H81 showed hardly any adhesion to the bar and a precise mapping of the bar surface within the concrete matrix. Thus, positive locking is the dominant force transmission mechanism (compare Figure 14a,b). In contrast, the pull-out specimens of HelixPul H83 demonstrated concrete adhesion to the rebar contour and, conversely, also exhibited damage to the CFRP contour due to material tearing (Figure 14c,d). This suggests the presence of an additional material bond between the concrete and the CFRP reinforcement.

In summary, the structural morphological investigations reveal that the majority of rebar variants transfer forces from the concrete to the reinforcement element primarily through form closure, rather than material closure. This form closure is primarily attributed to the geometry features, including the helical rib heights of the bars, the strong interlaminar shear strength within the bar, and the high surface quality. However, in cases where the bars exhibit roughness, including micro-roughness, as observed in the manufactured variant HelixPul H83, mineral crystals can grow into the bar structure. This phenomenon leads to an additional increase in bonding forces, further enhancing the effectiveness of force transfer between the concrete and the reinforcing elements.

Variation studies for the design of helix-pultruded bar contours as reinforcement elements in concrete were carried out for different geometry parameters. This determination was based on tensile tests and pull-out tests. The results highlight the significant potential for enhancing the bar´s bearing capacity and minimizing the manufacturing impact of the unique helix-pultrusion technique on the bar contours. This was determined by tensile tests and pull-out tests. Upon comparing the individual values obtained from these tests, it becomes evident that the HelixPul H85 variant exhibits the most favorable behavior compared to the other variants, marking it the recommended choice.

These findings confirm the initial assumption that novel helix pultrusion, as an efficient manufacturing process for carbon-reinforced bars, can achieve load-adapted and material-appropriate performance. Furthermore, this high potential can be further augmented through additional optimization efforts, such as employing higher-fiber volume contents. Additionally, the use of a thermoplastic matrix system allows for the flexible reshaping of the rebars, enabling adaption to more intricate structural configurations.

## 5. Conclusions and Outlook

The novel material—carbon–concrete composite—offers promising possibilities for applications in civil engineering. As part of the project preparation, the authors developed a novel production technology called helical pultrusion. This process enables the efficient and continuous production of surface-topologized rebars in a single process step, which are suitable for use as CFRP rebars in concrete applications. The presented geometric parameter adjustments and the produced rebars aim to enhance the load-bearing properties of the reinforcement elements and to overcome the current disadvantages of carbon–concrete-composite technology on the path towards series production:

- Reinforcing bar structures made of fiber composites enables thinner construction methods. In particular, carbon reinforcements are corrosion-resistant and reduce the required concrete cover.
- The newly developed helix-pultrusion process enables efficient manufacturing processes for fiber-composite reinforcement bar structures. It is characterized by few process steps and the effective use of materials. During the work, design parameters were varied, and corresponding variants were manufactured to achieve a more load-oriented material arrangement and utilization.
- The tensile behavior of the reinforcement bar prototypes was tested using a new, efficient tensioning system developed in-house. Compared to unfunctionalized reference bars, similar bar tensile strength was achieved with the contour adjustments, and the manufacturing influence of helix pultrusion was almost eliminated. The fracture strength of the HelixPul rebars was increased by more than 40% on average.
- By employing a more complex test arrangement, it was possible to determine torsional moment-free and transverse force-free pull-out characteristics, demonstrating good comparative pull-out behavior. The tensile force transmission in the composite for the HelixPul rebars was increased by more than a factor of 2.5.
- The determined preferred helix rebar contour, HelixPul H85, proves the suitability of these reinforcing bars from the novel production technology, due to element tension tests and concrete pull-out tests.

Through the geometry parameter variation of the HelixPul rebars, the potential and principle of helical pultrusion were successfully demonstrated. It allows the continuous production of reinforcing bars in a single-step forming process using carbon fibers with a thermoplastic matrix through pre-impregnated tapes. The transition to other fiber types and other thermoplastic matrix systems requires only minor adjustments. However, significant challenges in process technology and increasing productivity necessitate the integration of fiber plastic impregnation and in-situ polymerization into the process, along with increasing process speed. Implementing the manufacturing process with a thermoset matrix system requires major adaptations and novel semi-finished products. Therefore, further research is required to enhance the efficiency of the manufacturing process and utilize thermoset matrix systems for improved thermal resistance.

## Figures and Tables

**Table 1:** Overview of manufactured demonstrators of functionalized rods [19].

Functionalization	Abbreviation	Design Example
**Reference** **(no functionalization)**	RefNoFuc	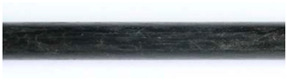
**Winding-forming by means of semi-finished tape**	WindForm	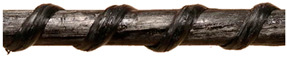
**Contour milling of** **functionalization**	ContMill	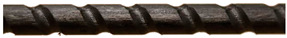
**Form-pressing of functionalization**	FormPres	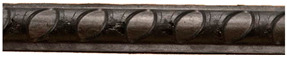
**Primary forming only with** **helix pultrusion**	HelixPul	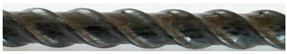
**Out-displacing and penetrating cylindrical elements**	ThermPin	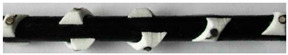

**Table 2:** Material characterization of unidirectional CF-PA6-Tape with Fiber-Volume-Ratio (FVR) of 0.45 and data sheet values [28,29].

Property	Measured Values (FVR = 0.45)	Data Sheet SGL CF-PA6_Tape * (FVR = 0.5)	Mixing Rule with Fiber Properties by SGL ** (FVR = 0.45)
Tensile Modulus E_1_	102 GPa	115 GPa	108 GPa
Tensile Strength F_tu;1_	1290 MPa	1800 MPa	1800 MPa
Elongation at Break A_1_	1.17%	1.48%	---
Tensile Modulus E_2_	101.6 GPa	---	---
Tensile Strength F_tu;2_	1290 MPa	---	---
Elongation at Break A_2_	3.05%	---	---
Compressive Strength R_s;1_	403 MPa	---	---
Compressive Strength R_s;2_	114.2 MPa	---	---
Shear Strength S_12_	64.2 MPa	---	---

(*) SGL TECHNOLOGIES GmbH—Datasheet: Unidirectional carbon fiber tape with thermoplastic matrix, status 08/2015; (**) SGL TECHNOLOGIES GmbH—Datasheet: SIGRAFIL Carbon-Endlosfasern, Status 02/2016.

**Table 3:** Geometric parameters of HelixPul geometry variants.

Design Variant	H81	H82	H83	H84	H85
**Geometry**	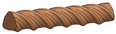	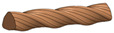	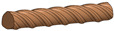	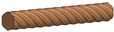	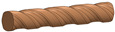
**Number of Helix Ribs *n***	3	3	3	6	2
**Pitch *P***	30 mm	60 mm	30 mm	30 mm	30 mm
**Inner Circle Diameter *D_i_***	7.3 mm	7.3 mm	7.3 mm	7.3 mm	7.3 mm
**Perimeter Diameter *D_p_***	9.3 mm	9.3 mm	8.3 mm	7.97 mm	8.5 mm
**Equivalent Diameter *D_e_***	8.0 mm	8.0 mm	7.57 mm	7.60 mm	7.97 mm
**Projected Flank Area *A_f_***	26.08 mm^2^	26.08 mm^2^	12.25 mm^2^	8.04 mm^2^	14.89 mm^2^
***A_f_*/*A_i_***	0.62	0.62	0.29	0.19	0.36

**Table 4:** Tensile test results of the HelixPul CFRP rebar variants H81, H83, H84 and H85.

Variant		F_max_ (kN)	σ_max_ (MPa)	ε_max_* (%)
**H81** ***n* = 6**	$\overset{-}{x}$	36.3	867.2	0.932
	***s***	1.6	38.4	0.134
	***ν* [%]**	4.43	4.42	14.36
**H83** ***n* = 6**	$\overset{-}{x}$	46.5	1111.8	0.912
	***s***	3.1	73.5	0.294
	***ν* [%]**	6.63	6.61	32.21
**H84** ***n* = 6**	$\overset{-}{x}$	53.2	1270.8	1.068
	***s***	1.6	38.1	0.163
	***ν* [%]**	3	3	15.27
**H85** ***n* = 6**	$\overset{-}{x}$	52.1	1245.2	1.19
	***s***	2.6	63.1	0.106
	***ν* [%]**	5.06	5.07	8.94

(*) Strain measurements not valid due to measuring issues.

**Table 5:** Pull-out test results of HelixPul CFRP rebar variants H81, H83 and H84 in concrete.

Variant		*F_max_* (kN)	*k_max_* (N/mm)
**H81** ***n* = 3**	$\overset{-}{x}$	3.98	179.02
	***s***	1.09	24.05
	***ν* (%)**	27.46	13.43
**H83** ***n* = 4**	$\overset{-}{x}$	9.28	325.57
	***S***	0.76	26.82
	***ν* (%)**	8.22	8.33
**H84** ***n* = 3**	$\overset{-}{x}$	7.00	237.19
	***s***	0.38	14.30
	***ν* (%)**	5.49	6.03

**Table 6:** Results of the RILEM-RC5 beam test of HelixPul CFRP rebar variants H81, H83, H84 and H85.

Variant		*F_max_* (kN)	∆*l_max_* (mm)	*f_max_* (mm)
**H81** ***n* = 3**	$\overset{-}{x}$	8.5	1.81	10.6
	***s***	1.38	1.14	4.89
**H83** ***n* = 4**	$\overset{-}{x}$	21.9	2.14	13.4
	***S***	0.63	0.44	0.94
**H84** ***n* = 4**	$\overset{-}{x}$	17.7	2.66	11.9
	***s***	1.49	0.2	0.77
**H85** ***n* = 2**	$\overset{-}{x}$	22.4	1.04	12.6
	***s***	4.63	0.36	3.1

**Table 7:** Comparison of the average fracture strengths in relation to the base material.

Variant (i)	$\overset{-}{x}$ = *σ_max_* (MPa)	*σ_max_*(*i*)/*σ_max_* (Ref)
RefNoFuc	1284.8	100.0%
ContMill	1239.8	96.5%
HelixPul H81	866.1	67.4%
HelixPul H83	1111.8	86.5%
HelixPul H84	1270.9	98.9%
HelixPul H85	1245.2	96.9%
FormPres	895.0	69.7%
WindForm	1163.2	90.5%
ThermPin	753.3	58.6%

## Data Availability

The generated data is stored and secured within the internal data management system of Technische Universität Dresden and is available on request.
